# Memantine for Refractory Obsessive-Compulsive Disorder: Protocol for a Pragmatic, Double-blind, Randomized, Parallel-Group, Placebo-Controlled, Monocenter Trial

**DOI:** 10.2196/39223

**Published:** 2023-05-11

**Authors:** Annalisa Maraone, Alessandro Trebbastoni, Antonella Di Vita, Fabrizia D'Antonio, Carlo De Lena, Massimo Pasquini

**Affiliations:** 1 Sapienza University of Rome Roma Italy; 2 Istituto di Ricovero e Cura a Carattere Scientifico Ospedale San Raffaele Rome Italy

**Keywords:** obsessive-compulsive disorder, refractory patients, antiglutamatergic agents, glutamate, augmentation medication, memantine, double blind, parallel group, placebo, OCD, psychiatric, distress, symptoms, neuropsychiatric, disability, overactivity, drug, treatment, Alzheimer, cognitive, titration, medication, mental health, psychiatric disorder

## Abstract

**Background:**

Obsessive-compulsive disorder (OCD) is a psychiatric syndrome characterized by unwanted and repetitive thoughts and repeated ritualistic compulsions for decreasing distress. Symptoms can cause severe distress and functional impairment. OCD affects 2% to 3% of the population and is ranked within the 10 leading neuropsychiatric causes of disability. Cortico-striatal-thalamo-cortical circuitry dysfunction has been implicated in OCD, including altered brain activation and connectivity. Complex glutamatergic signaling dysregulation within cortico-striatal circuitry has been proposed in OCD. Data obtained by several studies indicate reduced glutamatergic concentrations in the anterior cingulate cortex, combined with overactive glutamatergic signaling in the striatum and orbitofrontal cortex. A growing number of randomized controlled trials have assessed the utility of different glutamate-modulating drugs as augmentation medications or monotherapies for OCD, including refractory OCD. However, there are relevant variations among studies in terms of patients’ treatment resistance, comorbidity, age, and gender. At present, 4 randomized controlled trials are available on the efficacy of memantine as an augmentation medication for refractory OCD.

**Objective:**

Our study’s main purpose is to conduct a double-blind, randomized, parallel-group, placebo-controlled, monocenter trial to assess the efficacy and safety of memantine as an augmentative agent to a selective serotonin reuptake inhibitor in the treatment of moderate to severe OCD. The study’s second aim is to evaluate the effect of memantine on cognitive functions in patients with OCD. The third aim is to investigate if responses to memantine are modulated by variables such as gender, symptom subtypes, and the duration of untreated illness.

**Methods:**

Investigators intend to conduct a double-blind, randomized, parallel-group, placebo-controlled, monocenter trial to assess the efficacy and safety of memantine as an augmentative agent to a selective serotonin reuptake inhibitor in the treatment of patients affected by severe refractory OCD. Participants will be rated via the Yale-Brown Obsessive Compulsive Scale at baseline and at 2, 4, 6, 8, 10, and 12 months. During the screening period and T4 and T6 follow-up visits, all participants will undergo an extensive neuropsychological evaluation. The 52-week study duration will consist of 4 distinct periods, including memantine titration and follow-up periods.

**Results:**

Recruitment has not yet started. The study will be conducted from June 2023 to December 2024. Results are expected to be available in January 2025. Throughout the slow-titration period, we will observe the minimum effective dose of memantine, and the follow-up procedure will detail its residual efficacy after drug withdrawal.

**Conclusions:**

The innovation of this research proposal is not limited to the evaluation of the efficacy and safety of memantine as an augmentation medication for OCD. We will also test if memantine acts as a pure antiobsessive medication or if memantine’s ability to improve concentration and attention mimics an antiobsessive effect.

**Trial Registration:**

ClinicalTrials.gov NCT05015595; https://clinicaltrials.gov/ct2/show/NCT05015595

**International Registered Report Identifier (IRRID):**

PRR1-10.2196/39223

## Introduction

### Background

Obsessive-compulsive disorder (OCD) is one of the most disabling neuropsychiatric disorders, characterized by repetitive and intrusive thoughts (obsessions) and repeated ritualistic behaviors (compulsions) that are performed by affected individuals to decrease distress [1]. It is estimated that 2.5% to 3% of the general population are affected by OCD [2], which has some sex-related features [3]. Moreover, in the last decade, evidence suggests significant neuropsychological deficits—independent of severity, comorbidity, and medication—in patients with OCD [4,5]. Several studies suggest impairments in executive function, processing speed, sustained attention, and nonverbal memory [6-11].

Prior to the *Diagnostic and Statistical Manual of Mental Disorders, Fifth Edition* (DSM-5) [12], OCD was included as an anxiety disorder because patients commonly reported anxiety as a symptom [13]. However, in the DSM-5, OCD was moved to its own distinct section—*Obsessive-Compulsive and Related Disorders*—because the condition was considered to be more closely related to other disorders [14].

Typically, OCD is determined by obsessions (irrational beliefs) and compulsions that are rational avoidance responses activated by these irrational beliefs. Recent data [15,16] instead suggest that the mechanism underlying OCD can be found in an interference in the balance between the habit learning system and the goal-directed system. The habit learning system is founded on historical information, independent of past rewards, potentially resulting in behavioral inflexibility even in light of rapid changes in the environment. On the other hand, the goal-directed system applies control over habits in response to changes, including changes in response to the assessment of actions and outcomes. A study conducted by Voon and colleagues [17] found that patients with OCD made choices generally based on a model-free approach (habit learning system) rather than a model based on a goal-directed system.

In the current habit hypothesis of compulsivity, compulsions result from a deficit of goal-directed actions, rather than being driven by irrational beliefs. In line with this much more behavioral account of the disorder, OCD was inserted in the new *Obsessive-Compulsive and Related Disorders* chapter of the DSM-5.

Recurrent circuits that connect the cerebral cortex to the basal ganglia, which are termed *cortico-striatal-thalamo-cortical circuitry* (CSTC) *loops*, are involved in habits. CSTC dysfunctions, as structural abnormalities, alter brain activation and connectivity [18,19] and seem to be implicated in OCD. According to this hypothesis, the hyperactive or hyperconnected CSTC loops are self-exciting in a runaway positive feedback loop [20,21], resulting in the need to perform compulsions, which in turn reinforces habits and increases the urge to perform compulsions [22].

The primary neurotransmitter involved in the CSTC model of OCD is glutamate [23]; it also has a fundamental role in neuronal plasticity, learning, and memory [24]. Excessive levels of glutamate in the brain area can stimulate glutamate receptors, resulting in hyperactivity or even excitotoxicity in neurons; in pathological conditions, glutamate can act as a neuronal excitotoxin, resulting in rapid or late neurotoxicity [25].

In support of this hypothesis, a large mega-analysis and meta-analysis of imaging data found age-related changes in brain structures, suggesting that OCD is underpinned by complex, nonlinear neurodevelopmental mechanisms [26,27].

The pathophysiology of OCD indicates aberrant glutamatergic signaling, with reduced glutamatergic concentrations in the anterior cingulate cortex and increased glutamatergic concentrations in the striatum and orbitofrontal cortex [28]. Moreover, both ionotropic glutamate receptors and metabotropic glutamate receptors are linked in virtually every form of learning in the brain, including habit learning [15,29]; are localized in brain circuitry; and are candidates in the pathophysiology of OCD. Several studies show evidence of altered glutamate homeostasis in OCD, with higher levels of glutamate in the cerebrospinal fluid of patients with OCD compared to those of healthy controls [30,31]. Genetics studies suggest an association between polymorphisms of glutamate-associated genes and the development of OCD [32-34]; in particular, OCD in families has been associated with a polymorphism of the gene that codes for N-methyl-D-aspartic acid receptors [32].

Conventionally, guidelines suggest the use of medications that act on serotonergic pathways, such as serotonin reuptake inhibitors (SRIs) and, in particular, selective SRIs (SSRIs), for OCD. Even if the exact mechanisms of their actions remain mysterious, it seems that SRIs exert their effects by influencing CSTC [35]. Unfortunately, it is estimated that around 30% to 60% of patients with OCD do not attain remission after taking SRIs [5]; even when switching to another SSRI, clomipramine, or an atypical antipsychotic, around 30% of patients remain untreated for refractory symptoms.

Given the role that glutamate appears to play in the dysregulation of OCD, glutamatergic drugs could be potential candidates for an OCD therapy augmentation strategy. Glutamate-modulating drugs seem to be potential therapeutic agents for other psychiatric disorders, such as depression, bipolar disorder, and suicidality, that have high comorbidity with OCD [36-38].

In light of the new evidence, several authors have focused their interest on the use of glutamate-modulating agents in patients with refractory OCD. Indeed, some studies that used memantine; riluzole; ketamine; or nutritional supplements, such as inositol and N-acetylcysteine, suggested promising benefits for these kinds of patients [39].

Several randomized controlled trials (RCTs) on the effects of glutamate-modulating drugs on OCD were conducted. A review conducted by Marinova and colleagues [39] showed that memantine seems to have a positive effect on refractory symptoms as an augmentation medication for OCD.

Due to substantial differences in treatment resistance, comorbidity, age, and gender among patients [39], at the moment, it is premature to recommend the use of glutamatergic drugs for refractory symptoms, since more studies are needed to confirm the use of this pharmacological approach for OCD augmentation therapy [40]. Moreover, randomized placebo-controlled trials with larger study populations are necessary in order to draw definitive conclusions on the utility of glutamate-modulating drugs for OCD.

Identifying and confirming the efficacy and safety of new therapeutic approaches for OCD could also be very useful for the purpose of early intervention in patients. Even if there are not sufficient data to support a progression of brain changes, it is likely that early intervention could help to reduce damage resulting from neurotoxicity [41].

### Objectives

The main purpose of our study is to conduct a double-blind, randomized, parallel-group, placebo-controlled, monocenter trial to assess the efficacy and safety of memantine—a low- to moderate-affinity, noncompetitive N-methyl-D-aspartic acid receptor antagonist [40] that is currently approved for the treatment of Alzheimer disease—as an augmentative agent to an SSRI in the treatment of moderate to severe OCD. The second aim of the study is to evaluate the effect of memantine on the concentration and attention abilities of patients with OCD. The third aim is to investigate if responses to memantine could be modulated by variables such as gender, symptoms subtypes, and the duration of untreated illness.

## Methods

**Figure 1:**
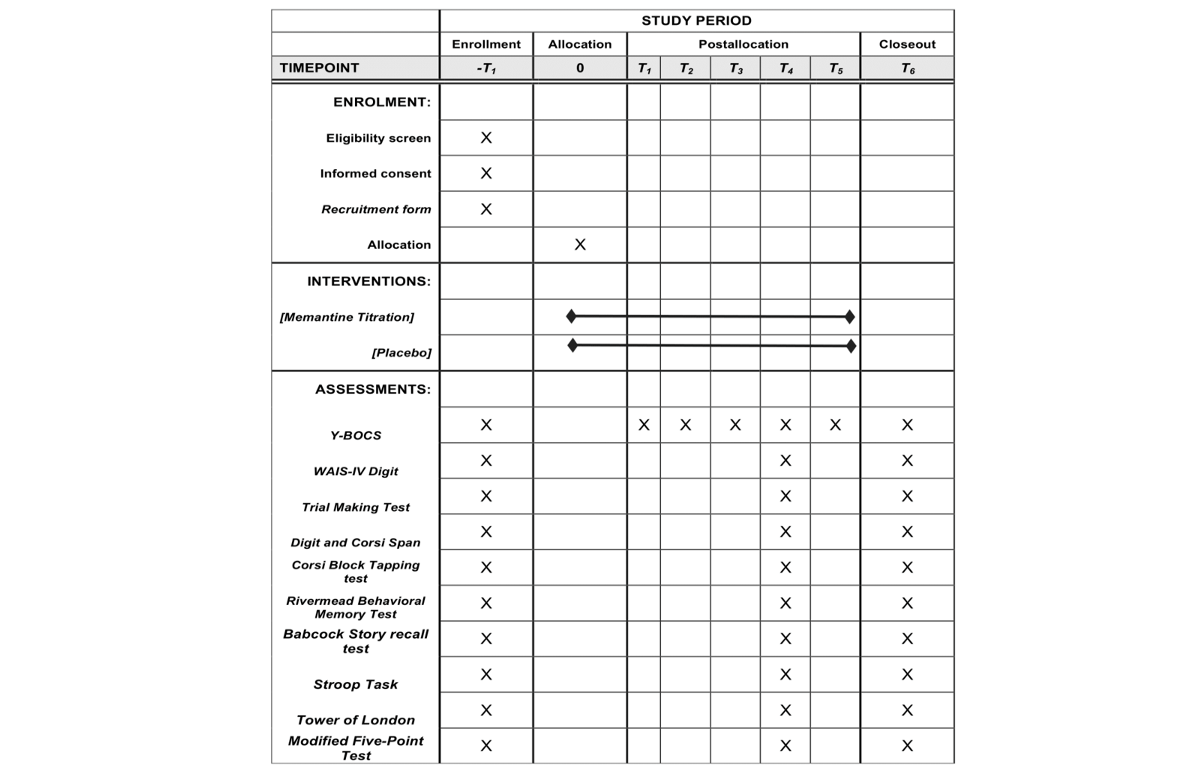
Spirit figure.

This protocol has been reported according to the SPIRIT (Standard Protocol Items: Recommendations for Interventional Trials) statement requirements (Figure 1). The complete SPIRIT checklist is available in Multimedia Appendix 1.

### Trial Design

A double-blind, randomized, parallel-group, placebo-controlled, monocenter trial will be conducted over 52 weeks at the outpatient clinic of the Department of Human Neurosciences, Sapienza University of Rome. The study population will include patients diagnosed with moderate to severe OCD according to the diagnostic criteria of the DSM-5 who consecutively access the OCD outpatient clinic of the Department of Human Neurosciences, Sapienza University of Rome. The investigators will be academic research assistants and psychiatrists with experience in OCD.

We will enroll patients who meet the diagnostic criteria of the DSM-5 [12] for moderate to severe OCD, and those with a Yale-Brown Obsessive Compulsive Scale (Y-BOCS) [42] score of >21 will be selected for inclusion in the study. The Y-BOCS, which will be used to evaluate treatment efficacy, is a well-validated, 10-item rating scale that is widely used to measure the severity of OCD symptoms. Participants will be rated with the Y-BOCS at baseline and at 2, 4, 6, 8, 10, and 12 months.

During the screening period and T4 and T6 follow-up visits, all participants will undergo an extensive neuropsychological evaluation. We will assess processing speed by using the Wechsler Adult Intelligence Scale, Fourth Edition, Digit Span [43]; attention by means of the Trial Making Test [44]; computerized alertness and go/no-go task memory by using the Digit and Corsi Span [45], a modified version of the Corsi Block-Tapping Test [46], the story recall portion of the Rivermead Behavioral Memory Test [47], and a modified version of the Babcock Story Recall Test [48]; and executive functions by means of the Stroop task [49], a modified version of the Tower of London test [50], and the Modified Five-Point Test [51].

### Ethical Considerations

The study will be conducted in accordance with the Declaration of Helsinki and its subsequent revisions. Written informed consent will be obtained from all eligible participants, following the complete description of study details. Participants will be informed about their freedom to withdraw from the trial anytime without any negative effect on their therapy. The study was approved for a grant by Sapienza University of Rome (grant RM11916B7477A1B6), approved by the local ethics committee (protocol number: 0784/2022), and registered on ClinicalTrials.gov (trial number: NCT05015595).

Only patients legally capable of giving their consent can participate in the study. The investigator will be responsible for the correctness of the recruitment procedure and will be asked to use comprehensible verbal communication when providing information to the patients. Before entering the study, patients will be fully informed about the purposes of the research; possible benefits; any potential, personal, reasonable risks or discomforts; and the expected duration of their participation in the trial. The investigator will also clearly inform the patients that they can leave the study at any time and for any reason, without giving an explanation, and that discontinuation would not, in any case, deteriorate the patients’ relationship with the physician or the possibility of receiving alternative therapies.

Before recruitment, a copy of the informed consent form and personal data processing consent form will be given to the patients, together with any needed clarification. Sufficient time will be given to enable the patients to decide whether they will participate in the study. Moreover, we intend to also seek approval from the Institutional Review Board of Sapienza University of Rome.

### Participants

The inclusion criteria are as follows: a psychiatrist diagnosis of current moderate to severe OCD based on the DSM-5, a Y-BOCS [42] score of >21, an age of 18 to 55 years, patients in therapy with a stable SSRI for at least 3 weeks prior to enrollment, and written informed consent.

The exclusion criteria are as follows: substance dependence, an IQ of <70, comorbid psychiatric disorders, pregnant or breastfeeding women or women who intend to become pregnant during the period of the study, and concomitant treatments (repetitive transcranial magnetic stimulation, cognitive behavioral therapy, or other glutamate-modulating drugs).

### Interventions

This is a protocol for a double-blind, randomized, parallel-group, placebo-controlled, monocenter trial in patients with OCD. The trial will include 1 active memantine dose arm and 1 placebo arm.

**Figure 2:**
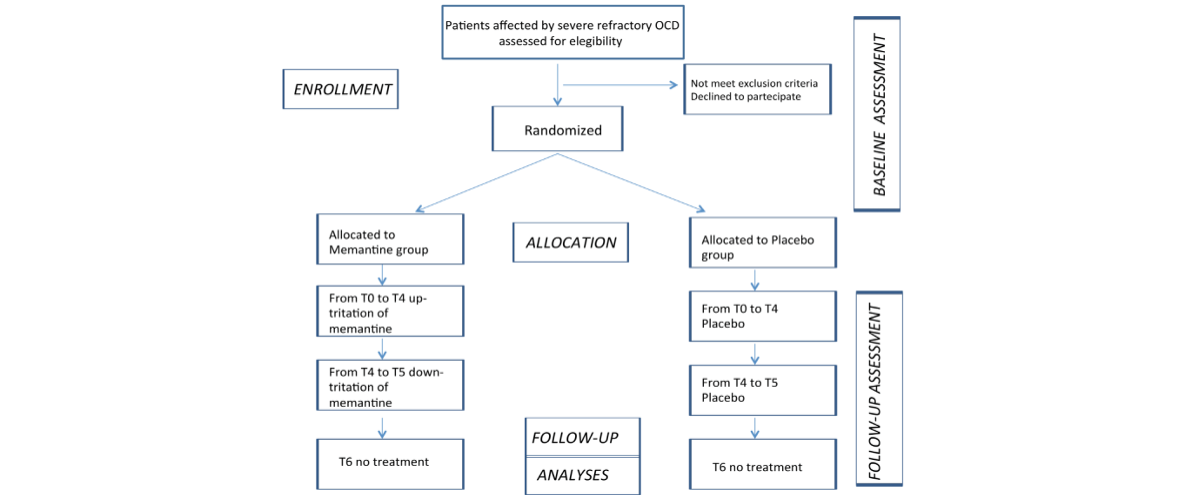
Study Flow-chart.

**Figure 3:**
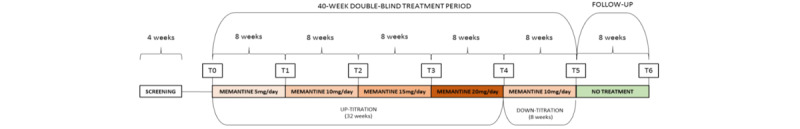
Study Timeline.

The trial will consist of the following four distinct periods:

- Screening: the screening period may last up to 4 weeks for each eligible patient.
- A 32-week, double-blind, up-titration treatment period (from T0 to T4): after screening, patients who meet all eligibility criteria will be randomly assigned to 1 of 2 arms (memantine or placebo) in a 1:1 ratio. Following baseline assessments, each patient will receive a daily dose of memantine or the placebo (up to 20 mg/day).
- An 8-week, double-blind, down-titration treatment period (from T4 to T5): at T4, the dose of memantine or the placebo will be reduced to 10 mg per day due to safety reasons, before the end of treatment (T5).
- Follow-up period (from T5 to T6): after the 40-week, double-blind treatment period, patients will be asked to come back for the follow-up visit (T6) 8 weeks after the end of the treatment (T5; Figures 2 and 3).

### Recording of Adverse Events

The investigator will carefully monitor the patients’ symptoms and signs, which will be either spontaneously reported or detected, throughout the whole study period up to the last visit scheduled in the study protocol. On a case-by-case basis, a prolonged observation period may be requested.

Adverse events that occur during the clinical trial will be assessed by the investigator in terms of their seriousness and relationship with the investigational product, and the principal investigator will be notified.

As soon as a severe adverse event occurs, an ad hoc form for severe adverse events will be completed, in accordance with the European Union regulation about pharmacovigilance in clinical research [52]. If, for any reason, the disadvantages of participation appear to be significantly greater than those foreseen, the investigator will inform the principal investigator and the bodies providing ethical oversight to evaluate trial discontinuation for the patient.

### Sample Size

The sample size was calculated by using the G*Power 3.1.9.2 software (Heinrich-Heine-Universität Düsseldorf) [53]. By using the ANOVA repeated measures test with a within-between group interaction approach (considering changes in Y-BOCS scores as the primary outcome), an α of .5, a power (1 − β) of 0.85, 2 groups (SSRI and memantine group vs SSRI and placebo group), and 7 measurements (T0 to T6); and by assuming a correlation of 0.5 among repeated measures and a nonsphericity correction ε of 1, we determined that a total sample size of 20 participants (10 in each group) will be necessary to achieve a moderate effect size (*f*=0.25).

### Randomization and Blinding

Placebo and memantine tablets will be identical in shape, color, weight, and scent. We will prepare in advance a computer-generated random number sequence for random group assignment. Treatment allocation will be concealed from the patients, from the physicians rating the patients, and from the statistician. Separate individuals will be responsible for randomization and the rating of patients.

### Data Collection and Quality Control

Patients will be enrolled after the physician in charge and a supervisor check the patient information regarding the inclusion and exclusion criteria. The data of all participants, including those who are discontinued or dropped from intervention protocols, will be collected according to the study protocol. Quality control will be performed by an external data manager (recruited exclusively for this project).

### Statistical Methods

Descriptive statistics for baseline characteristics and the measurements at each time point will be calculated. For the primary objective, a repeated measures ANOVA approach will be used to detect between- and within-group differences in Y-BOCS variables. *P* values of <.05 will be considered statistically significant. Data will be analyzed by using SPSS version 20 (IBM Corp).

## Results

Recruitment will start in June 2023, and data collection is expected to end in 2024. Results are expected to be available in January 2025. We also intend to make publications available in 2025, and the results of our trial will also be presented at conferences.

## Discussion

### Overview

In light of the possible role of glutamatergic signal dysregulation in OCD, it is possible to find, among glutamatergic drugs, candidates for an OCD therapy augmentation strategy.

This has motivated the use of glutamate modulators in patients who are unresponsive to standard pharmacotherapeutic approaches. Several RCTs that used glutamate-modulating agents, such as memantine, riluzole, N-acetylcysteine, and glycine, reported promising benefits [38,54,55]. In particular, 4 RCTs were conducted that used memantine (vs a placebo) as an augmentative agent to SRIs in the treatment of moderate to severe OCD [56].

Marinova and colleagues [39] reported that memantine was the compound that most consistently showed a positive effect, as an augmentation medication, on OCD in their study. A critical letter [40] exposed some concerns on this conclusion and proposed that, in light of some methodological gaps, it is necessary to conduct more studies on the use of memantine as an OCD augmentation therapy.

### Limitations

Our outpatient clinic—a center specializing in OCD—is an academic service of the Department of Human Neurosciences, Sapienza University of Rome. This could represent a limitation—patient selection bias—due to patients’ access to the center’s services. Moreover, our study will use a small sample size due to the limits of service access and the inclusion criteria.

### Conclusion

To the best of our knowledge, only 4 RCTs have been conducted in recent years that investigated the effects of memantine in patients with refractory severe OCD; our trial will be the first one of its kind in Italy. The innovation of this research proposal is not limited to the evaluation of the efficacy and safety of memantine as an augmentation medication for OCD. Through slow titration and follow-up, we will also observe the minimum effective dose of memantine and its residual efficacy after drug withdrawal by using an 8-week follow-up procedure. In addition, by measuring potential executive function impairments during the study period, we might confirm whether memory and attention are modulators of overall clinical improvement. In other words, we will test if memantine, being an antiglutamatergic agent, acts as a pure antiobsessive medication or if memantine’s ability to improve concentration and attention mimics an antiobsessive effect.

## Appendix

Multimedia Appendix 1 SPIRIT (Standard Protocol Items: Recommendations for Interventional Trials) checklist.

## Abbreviations

CSTC: cortico-striatal-thalamo-cortical circuitry
DSM-5: *Diagnostic and Statistical Manual of Mental Disorders, Fifth Edition*
OCD: obsessive-compulsive disorder
RCT: randomized controlled trial
SPIRIT: Standard Protocol Items: Recommendations for Interventional Trials
SRI: serotonin reuptake inhibitor
SSRI: selective serotonin reuptake inhibitor
Y-BOCS: Yale-Brown Obsessive Compulsive Scale

## Data Availability

The data generated and analyzed during this study will be available from the corresponding author on reasonable request, following the completion of the trial and publication of the main outcomes paper, and will be included in any published articles.
