# Supplementary material for: Memantine for Refractory Obsessive-Compulsive Disorder: Protocol for a Pragmatic, Double-blind, Randomized, Parallel-Group, Placebo-Controlled, Monocenter Trial
Source: JMIR Res Protoc. 2023 May 11;12:e39223. doi: 10.2196/39223 (PMC10214117; doi:10.2196/39223)
Supplement: Multimedia Appendix 1 [file resprot_v12i1e39223_app1.pdf]

SPIRIT

STANDARD PROTOCOL ITEMS: RECOMMENDATIONS FOR INTERVENTIONAL TRIALS

SPIRIT 2013 Checklist: Recommended items to address in a clinical trial protocol and related documents*

Section/item	Item No	Description
**Administrative information**		
Title	1	Memantine for refractory Obsessive-Compulsive Disorder: Protocol for a pragmatic double blind, randomized, parallel group, placebo controlled, monocenter trial
Trial registration	2a	ClinicalTrials.gov: NCT05015595 (MemaROCD)
Protocol version	3	Registred on 20 August 2021
Funding	4	Sapienza University of Rome
Roles and responsibilities	5a	Department of Human Neurosciences, Sapienza University of Rome
	5b	Prof. Massimo Pasquini, M.D., PhD<br>Full Professor of Psychiatry
	5c	massimo.pasquini@uniroma1.it<br>Tel number: +39 06 49914121
	5d	A.M, M.P., A.T. and C.dL. have contributed to the conception and design of the research; A.M and M.P. will evaluete the patients; A.D.V. and F.D. will assign the patients to the two groups.A.M and M.P. have drafted the manuscript. All authors critically revised the manuscript, agree to be fully accountable for ensuring the integrity and accuracy of the work, and read and approved the final manuscript.
**Introduction**		
Background and rationale	6a	A growing number of RCTs have assessed the utility of different glutamate-modulating drugs as an augmentation or monotherapy in OCD, including refractory patients.
	6b	Placebo

Objectives	7	The main purpose of this study is to conduce a trial to assess the efficacy and safety of memantine as an augmentative agent to a SSRI in treatment of moderate to severe OCD. The second aim of the study is to evaluate the effect of memantine on cognitive funtions in OCD patients. The third aim is to investigated if response to memantine could be modulate by variables such as gender, symptoms subtypes and duration of untrated illness.
Trial design	8	A double blind, randomized, parallel group, placebo controlled, monocenter trial

Methods: Participants, interventions, and outcomes

Study setting	9	Outpatient clinic of the Department of Human Neurosciences, Sapienza University of Rome (academic hospital)
Eligibility criteria	10	Inclusion criteria: diagnosis by a psychiatrist of current moderate to severe OCD according to the DSM 5, Yale Brown Obsessive Compulsive Scale (Y-BOCS) [42] score of >21, age between 18 to 55 years; patients in therapy with a stable SSRI for at least three weeks prios; written informed consent.<br>Exclusion criteria: Substance dependence, IQ <70, comorbid psychiatric disorders; female pregnant or breast-feeding or intend to become pregnant during the period of the study; concominant treatments (rTMS, CBT, other glutamate-modulating drugs).
Interventions	11a	This is a double blind, randomized, parallel group, placebo controlled, monocenter trial in subjects with OCD. The trial includes one active dose arm of Memantine and placebo.<br>The trial consists of four distinct periods:<br>• Screening: The screening period may last up to 4 weeks for each eligible patient.<br>• Thirty-two-week double-blind up-titration treatment period (from T0 to T4): After screening, patients who meet all eligibility criteria will be randomly assigned to one of two arms (Memantine or placebo) in a 1:1 ratio. Following baseline assessments, each patient will receive a daily administration of Memantine/placebo up to 20mg/day.<br>• Eight-week double-blind down-titration treatment period (from T4 to T5): At T4, the dose of Memanntine/placebo will be reduced at 10mg/day due to safety reasons before the end of treatment (T5).<br>• Follow-up period (from T5 to T6): After the 40-week double-blind treatment period, patients will be asked to come back for the follow-up visit (T6) 8 weeks after the end of the treatment (T5).

	11b	As soon as a severe adverse event occurs, an ad hoc form for severe adverse events will be completed, in accordance with the EU regulation about pharmacovigilance in clinical research [52]. If for any reason the disadvantages of participation appear to be significantly greater than foreseen, the investigator will inform the principal investigar and the bodies providing ethical oversight to evaluate to evaluate trial discontinuation for the patient.
	11c	The Investigator will also clearly inform the patient that she can leave the study at any time and for any reason without giving an explanation, and that this discontinuation would not in any case deteriorate the patient's relationship with the physician and/or the possibility of receiving alternative therapies.
	11d	concomitant use of SSRI are permitted<br>concomitant use of rTMS, CBT, other glutamate-modulating drugs are prohibited during the trial
Outcomes	12	Participants were rated by Y-BOCS at baseline and at 2, 4, 6, 8, 10 and 12 months.<br>During the screening period, T4 and T6 follow-up visits all participants will undergo an extensive neuropsychological evaluation. Speed processing by using the WAIS-IV Digit [43] attention by means of the Trial Making Test [44] and a computerized alertness and Go/no go task memory by using the Digit and Corsi Span [45] , a modified version of Corsi Block Tapping test [46], the Story recall of Rivermead Behavioral Memory Test [47] and a modified version of Babcock Story recall test [48] executive functions by means of Stroop Task [49], a modified version of Tower of London [50] and the Modified Five Point Test [51].
Participant timeline	13	Study design consists of four distinct periods (52 weeks) including memantine titration and follow-up. (see Figure 1 and 2)
Sample size	14	The sample size has been calculated using the GPower 3.1.9.2 software [53]. Using the ANOVA repeated measures test a with a within-between groups interaction approach (considering changes in YBOCS scale as primary outcome), with alpha=0.5, power (1-beta)=0.85, with 2 groups (SSRI + memantine vs SSRI + placebo) and 7 measurements (T0 – T6), and assuming a correlation among repeated measures=0.5 and a nonsphericity correction epsilon=1, a total sample size of 20 participants (10 in each group) will be necessary to achieve a moderate effect size (f=0.25).
Recruitment	15	Strategies for achieving adequate participant enrolment to reach target sample size

Methods: Assignment of interventions (for controlled trials)

Allocation:

Sequence generation	16a	In advance we will prepared a random-number sequence that a computer-generated for random group assignment
Allocation concealment mechanism	16b	Mechanism of implementing the allocation sequence (eg, central telephone; sequentially numbered, opaque, sealed envelopes), describing any steps to conceal the sequence until interventions are assigned
Implementation	16c	Treatment allocation will be concealed from patients and physician will rated patients and from statistician.
Blinding (masking)	17a	Separate individuals will be responsible for randomization and rating patients.
	17b	In case a severe adverse events, related to trial, will be occurrent the unblinding is permissible

Methods: Data collection, management, and analysis

Data collection methods	18a	Participants were rated by Y-BOCS at baseline and at 2, 4, 6, 8, 10 and 12 months. During the screening period, T4 and T6 follow-up visits all participants will undergo an extensive neuropsychological evaluation
Data management	19	Patients will be enrolled after the doctor-in- charge and a supervisor check the patient information regarding the inclusion and exclusion criteria. The data of all participants including those who will be discontinued or dropped from intervention protocols will be collected according to study protocol. Whereas the quality control will be performed by a external data manager (recruited exclusively for this project).
Statistical methods	20a	Descriptive statistics for baseline characteristics and the final measurement at each time point will be calculated. A repeated measures will be used to detect the statistical significance of overall differences across the psychological variables when comparing the two groups. Test ANOVA will be used for multivariate correlation. All p-values =0.05 were considered statistically significant. Data were analyzed using SPSS version 13.0.

Methods: Monitoring

Data monitoring	21a	A.D.V. and F.D. will assign the patients to the two groups and monitoring the study.

Harms	22	Adverse events occurred during the clinical trial will be assessed by the Investigator in terms of seriousness and relationship with the investigational product and will be notified to principal investigator and local ethics committee.
**Ethics and dissemination**		
Research ethics approval	24	local ethics committee (Prot. 0784/2022)
Protocol amendments	25	/
Consent or assent	26a	Only patients legally capable of giving their consent can participate in the study. The Investigator is responsible for the correctness of the recruitment procedure and is asked to use a comprehensible verbal communication in providing information to the patients. Before entering the study, patients will be fully informed about the purposes of the research, possible benefits, any potential personal reasonable risk or discomfort, the expected duration of their participation in the trial.
	26b	/
Confidentiality	27	At baseline, during the trial, follow up and after socio-demographic and clinical information will be collected along with the administration af the aforementioned validated rating scales.
Declaration of interests	28	All authors declare the absence of conflict of interests related to the present study.
Access to data	29	The data generated and analyzed during this study will be available from the corresponding author on reasonable request following the completion of the trial and publication of the main outcomes paper and will be included in any published articles.
Ancillary and post-trial care	30	/
Dissemination policy	31	/
**Appendices**		
Informed consent materials	32	Before recruitment, a copy of the informed consent form and personal data processing consent form will be given to the patient, together with any needed clarification.
Biological specimens	33	/

*It is strongly recommended that this checklist be read in conjunction with the SPIRIT 2013 Explanation & Elaboration for important clarification on the items. Amendments to the

protocol should be tracked and dated. The SPIRIT checklist is copyrighted by the SPIRIT Group under the Creative Commons "Attribution-NonCommercial-NoDerivs 3.0 Unported" license.
